# Enhanced Actuation Stability of Liquid Crystal Elastomer Actuators With Diels–Alder Dynamic Bonds

**DOI:** 10.1002/smll.74705

**Published:** 2026-08-02

**Authors:** Konstantinos Stamatis, Yoo Jin Lee, Asaf Dana, Manivannan Sivaperuman Kalairaj, Caleb A. Crusco, Jared A. Gibson, Sasha M. George, Svetlana A. Sukhishvili, Taylor H. Ware

**Affiliations:** ^1^ Department of Materials Science and Engineering Texas A&M University College Station Texas USA; ^2^ Department of Biomedical Engineering Texas A&M University College Station Texas USA; ^3^ Air Force Research Laboratory Wright Patterson Air Force Base Dayton Ohio USA; ^4^ AeroVironment, Inc. Fairborn, Dayton Ohio USA

**Keywords:** Diels–Alder bonds, dynamic diffraction grating, liquid crystal elastomers, reprocessing

## Abstract

Polymeric materials capable of undergoing reversible shape change can be used as soft actuators to create low‐density, compliant machines. Liquid crystal elastomers (LCEs) are a promising class of soft actuators that undergo large, reversible shape change by heating and cooling, but the fabrication of these materials requires molecular alignment to be trapped by crosslinking. LCEs with dynamic crosslinks can dramatically simplify materials processing and allow for reprogramming of the material after synthesis. However, thermally labile dynamic crosslinks can break during actuation, leading to a degradation of actuator performance over time. Herein, we developed LCEs with dynamic crosslinks with well‐separated actuation and processing temperature regimes. Diels–Alder (DA) crosslinks based on 3‐substituted furans are more thermally stable, achieving an actuation strain of 35.2% ± 0.5% and an actuation stress of 213.7 ± 20.1 kPa. These values are higher than those obtained from DA bonds formed with 2‐substituted furans, which have been commonly used in LCE systems with DA crosslinks. The introduction of 3‐substituted furan allowed reversible actuation and improved thermal stability relative to 2‐substituted furan, while maintaining reprogrammability and reprocessability. We leverage the combination of thermal stability and facile reprocessability to create multiple, dynamic diffraction gratings in a single material.

## Graphical abstract

By leveraging furan substitution, enhanced thermal stability during actuation of liquid crystal elastomers (LCEs) with Diels–Alder dynamic (DA) bonds was achieved. This work demonstrates that 3‐substituted furan broadens the actuation window of LCEs with DA bonds and enables superior thermomechanical stability and cycling performance at elevated temperatures relative to 2‐substituted furan.

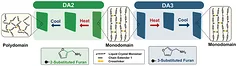

## 1 Introduction

Soft actuators can change shape and perform work on their environment in response to external stimuli. Unlike traditional rigid actuators, soft actuators are composed of compliant materials and, as a result, have received substantial attention in the fields of soft robotics [1, 2, 3, 4] and active medical devices [5, 6]. Thermally responsive polymer networks are particularly promising candidates for use as soft actuators because of their capability to undergo large, reversible deformations in response to external stimuli [7]. Semicrystalline shape memory polymers (SMPs) [8], hydrogels [9], and liquid crystal elastomers (LCEs) [10] are polymeric materials capable of achieving reversible actuation. Among those types of polymers, LCEs are unique in their ability to undergo the largest reversible shape changes without requiring mass transport [11]. Specifically, LCEs can generate actuation stresses of over 2 MPa [10, 12, 13] and strains of tens of percent relative to their original dimensions [10, 14] over at least 15 000 actuation cycles [15, 16].

The reversible shape change of LCEs originates from coupling of an order‐disorder phase transition and polymer chain conformation. To program an LCE actuator, rod‐like molecules (mesogens) need to be aligned and fixed through crosslinking, which results in an anisotropic polymer chain conformation [10, 17]. The Finkelmann method is a two‐step mechanical alignment process and was the first reported method for aligning LCEs. The first step involves partially crosslinking the material, followed by applying a constant load to achieve uniform alignment. In the second step, the network is fully crosslinked, and the alignment is locked [18]. This process has been adapted for use in a wide range of chemistries [19]. The presence of permanent crosslinks in these LCEs locks the material into a fixed orientation, preventing any reprogramming or reprocessing after the initial synthesis [17, 20, 21].

LCEs with dynamic crosslinks, rather than permanent ones, have emerged as a promising alternative for soft actuators that can be reprogrammed and reprocessed [22]. While conventional LCE actuators need to be aligned before the full formation of the permanent network, LCEs with dynamic bonds can be programmed via a post‐crosslinking alignment method, which is simpler than the traditional Finkelmann method and other variations of this method relying on two‐stage crosslinking [22]. Critically, dynamic crosslinks allow for reprogramming of the LCE [17]. LCEs with dynamic crosslinks can be realized by synthesizing thermoplastic LCEs with physical crosslinks [23, 24, 25], or LCEs with dynamic covalent bonds, which exchange via associative or dissociative mechanisms [17]. Dynamic associative bonds can be introduced within LCEs via ester‐based [22], carbamate‐based [26], boronic ester‐based [27], or siloxane‐based [28] crosslinks that exchange at elevated temperatures [22, 26, 28]. Dynamic associative bonds can also be introduced within LCEs by light‐induced radical‐mediated addition‐fragmentation chain transfer [29]. However, while LCEs with associative dynamic bonds can be reprogrammed, their reprocessing is difficult due to the largely maintained crosslink density and associated material viscosity [17, 22, 26, 28]. In contrast, LCEs with dissociative dynamic bonds, such as Diels–Alder (DA) bonds and spiral acetal bonds show a stronger reduction in crosslink density with temperature and are more readily reprocessable using typical melt and solvent processing methods [17, 30, 31]. DA bonds are a type of dissociative, dynamic covalent bond based on a catalyst‐free, “click,” thermoreversible [4 + 2] cycloaddition reaction [32] between furan (a conjugated diene) and maleimide (a dienophile) [33]. Thus, DA bonds allow for reprocessing either through complete bond dissociation at elevated temperatures or via lower temperature solid‐state plasticity involving reversible bond exchange within the network [32, 33, 34]. The incorporation of DA bonds in LCEs has been used to enable alignment after network formation [35, 36, 37], enabling the reprogramming and recycling of LCE actuators.

Despite the processing advantages of LCEs with DA bonds, loss of alignment during thermal actuation is an important challenge. Temperature plays a dual role by actuating the LCE while simultaneously dissociating a fraction of dynamic bonds, potentially compromising material integrity over time [17]. Ideally, to develop reprocessable actuators with performance comparable to conventional LCEs, the crosslinks should prevent loss of alignment during actuation, while allowing the greatest extent of material realignment and reprocessing at higher temperatures. All previous studies on LCEs with DA bonds have employed chemistry featuring 2‐alkyl‐substituted furan moieties. The DA bonds formed by the 2‐substituted furan rings and maleimide moieties start dissociating at relatively low temperatures of 55°C–65°C and become fully dissociated at temperatures around 110°C–120°C [30, 35, 36, 37, 38, 39]. The broad temperature range of dissociation of DA bonds (*T*_rDA_) overlaps with the broad nematic‐to‐isotropic transition temperature (*T*_NI_), resulting in severe LCE performance degradation due to the decreased number of crosslinks at elevated temperatures. One approach to address this limitation is to reduce T_NI_. T_NI_ can be tuned by diluting mesogen content or changing the intermolecular interactions between mesogens [38, 40, 41, 42, 43]. There are several limitations to relying solely on this approach. To avoid the dissociation of DA bonds, an actuator with a *T*_NI_ near room temperature would be needed. This would render the material sensitive to temperature changes in the local environment and reduce its potential use. This is further complicated by the broad nature of *T*_NI_ in polymer networks. Alternatively, this thermal stability challenge could be addressed by increasing the dissociation temperature of DA bonds.

The increase in dissociation temperature of DA bonds could provide a tool in mitigating alignment loss in LCE with DA bonds during thermal actuation. By exploiting regiochemistry in the furan ring, which refers to the preferential substitution of the methanamine group at specific positions on the furan ring, the dissociation temperature of DA bonds can be controlled [44]. Both theoretical and experimental studies on small‐molecule DA adducts with 3‐substituted furans further show higher thermal stability relative to their 2‐substituted counterparts [45, 46, 47]. The superior thermal stability of the 3‐substituted DA adducts is attributed to differences in electron distribution across the furan ring [45, 46, 47]. The mechanical strength and resistance to force induced dissociation of DA bonds with 3‐substituted furans in hydrogels were also superior to those of 2‐substituted analogs [48]. While 3‐substituted furans were incorporated within amorphous covalent adaptable networks to enhance their thermal stability and enable advantageous shape morphing properties [44], the effect of 3‐substituted furans on LCEs with DA bonds remains unexplored.

Here, we report the first introduction of a 3‐substituted furan into LCEs with DA bonds (DA3). We characterize the thermomechanical and shape morphing properties of DA3 compared to 2‐substituted furan analogs (DA2). Taking advantage of DA3's programmability, reprocessability, and thermomechanical robustness, we developed a reprogrammable actuator micropatterned with an integrated, dynamic diffraction grating.

## 2 Results and Discussion

### 2.1 Synthesis of DA3 and DA2 LCE Networks

**FIGURE 1:**
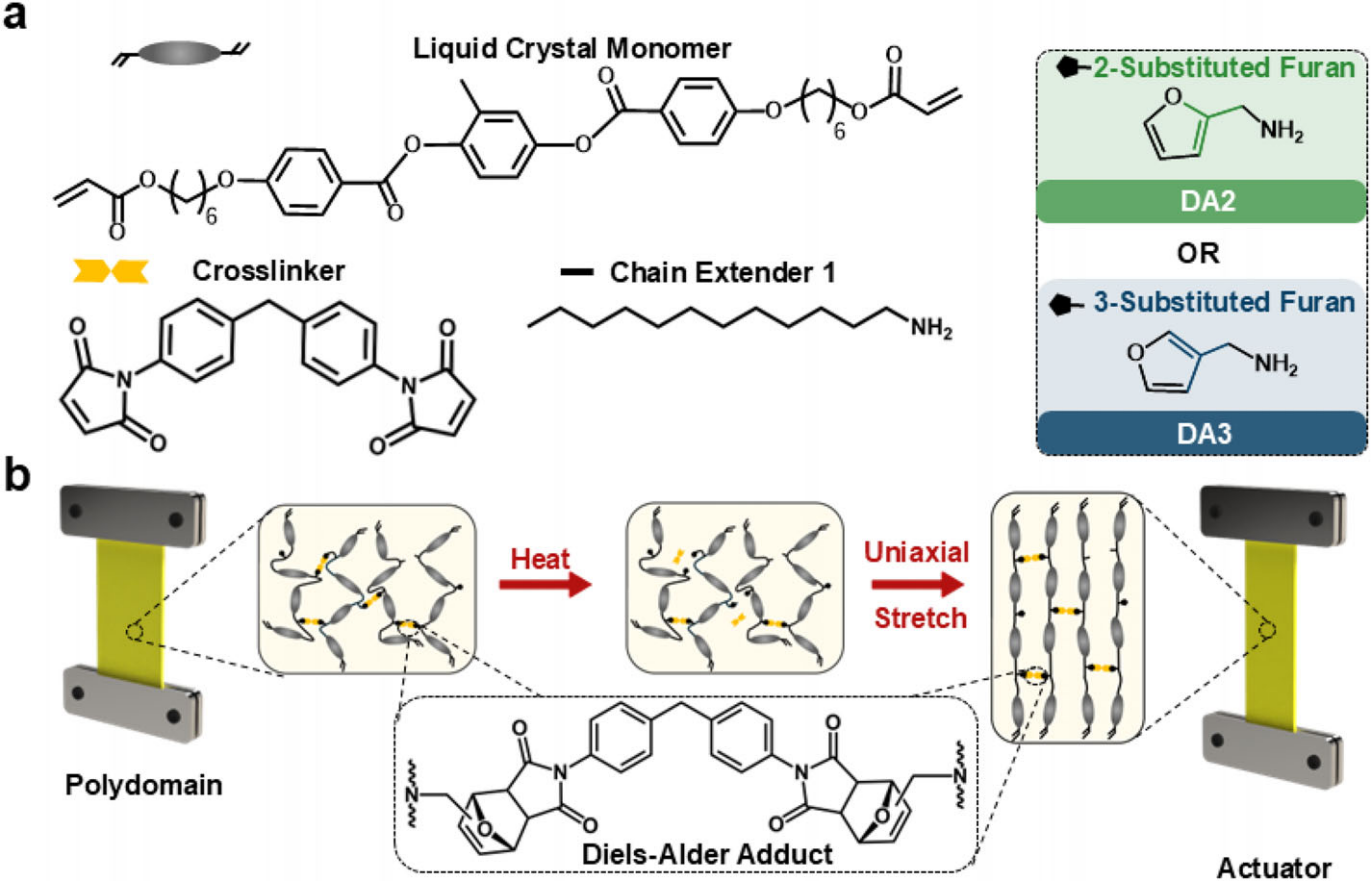
(a) The molecular structures of liquid crystal monomer (RM82), chain extender 1 (dodecan‐1‐amine), 2‐substituted furan (furan‐2‐ylmethanamine), 3‐substituted furan (furan‐3‐ylmethanamine), and crosslinker (1,1′‐(Methylenedi‐4,1‐phenylene)bismaleimide) for the synthesis of DA2 and DA3, (b) alignment of LCEs with DA bonds.

The effect of the regiochemistry of the furan ring on LCEs with DA bonds was investigated by synthesizing LCEs containing 3‐substituted furan (DA3) and comparing them to LCEs with 2‐substituted furan (DA2). Both DA2 and DA3 networks were synthesized using the same components: diacrylate nematic monomer, an aliphatic primary amine, a furan‐containing primary amine, and a bismaleimide (BMI) crosslinker (Figure 1). The only difference between DA2 and DA3 was the primary amine furans used for network synthesis, that is, DA2 incorporated 2‐substituted furan (furan‐2‐ylmethanamine), while DA3 utilized 3‐substituted furan (furan‐3‐ylmethanamine). Initially, linear polymeric precursors for LCEs were prepared by an acrylate‐amine Michael‐addition reaction between the diacrylate monomer RM82, an n‐alkyl primary amine, and primary amine containing a furan group. Nominally, we chose an acrylate‐to‐amine molar ratio of 1.1:1 and a furan‐to‐dodecylamine molar ratio of 0.8:0.2 (Figure S1,S2). The synthesis of the polymeric precursors was confirmed by comparing the integrated peak ratios of the proton signals from the acrylate group, the X‐substituted furan (X: 2 or 3), and dodecylamine. The theoretical furan‐to‐dodecylamine ratio was 4. The experimentally obtained furan‐to‐dodecylamine ratio was 3.3:1 for the polymeric precursor with the 2‐substituted and 3‐substituted furan. The ratio was determined from the relative integrals of the peaks corresponding to the two furan protons of the X‐substituted furan (b protons) and the three methyl protons of the dodecylamine chain end (c protons), respectively (Figure S3a,b). The resulting oligomers were solvent‐blended with 1,1′‐(methylenedi‐4,1‐phenylene)bismaleimide (BMI). During solvent evaporation, the furan‐functionalized precursors were crosslinked with BMI via the DA reaction, and polydomain DA2 and DA3 films were obtained. The polydomain films of LCEs were aligned via mechanical alignment by simply stretching on a tensile tester at temperatures near *T*_rDA_, followed by cooling to room temperature (Figure 1b).

The chemistry of the LCEs with DA bonds was selected to control the *T*_NI_ and *T*_rDA_. Specifically, the liquid crystallinity originates from the mesogenic diacrylate monomer (1,4‐bis[4‐(6‐acryloyloxyhexyloxy)benzoyloxy]‐2‐methylbenzene). N‐alkyl primary amines, such as dodecylamine, allow for the *T*_NI_ of the network to be tuned. The primary amine with the furan group has a dual role: it reacts with BMI, the crosslinker, through the DA reaction and controls the *T*_rDA_ of LCEs with DA bonds depending on the substitution of the furan.

### 2.2 Thermal Properties of DA2 and DA3

**FIGURE 2:**
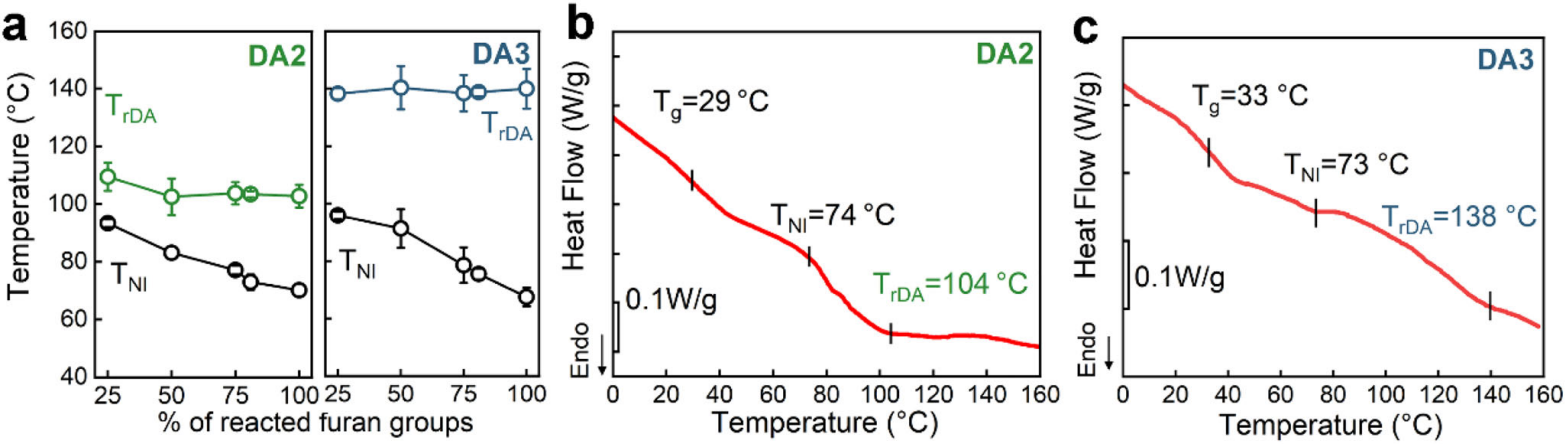
(a) Nematic‐to‐isotropic transition temperature (*T*_NI_) and retro‐DA dissociation temperature (*T*_rDA_) for DA2 and DA3 with different percentages of reacted furan groups. (b) Representative DSC curve of the second heating cycle for DA2. (c) Representative DSC curve of the second heating cycle for DA3. Each test was repeated 3 times. Each data point represents the mean (*n* = 3), and the error bars represent the standard deviation.

Heating LCEs with DA crosslinks above the *T*_NI_ to cause shape change may also trigger dissociation of a fraction of the DA bonds (Figure S2). Thus, the separation between *T*_NI_ and *T*_rDA_ is essential for the actuation performance and thermal stability of the LCEs with DA bonds. Decreasing *T*_NI_ at constant *T*_rDA_ can be used to enhance the separation between the transitions [38]. By increasing the length of the n‐alkyl amine chain extenders, the *T*_NI_ decreases for both DA2 and DA3 (Figure S4a), as previously reported [40, 49]. The decrease in the *T*_NI_ is observed due to the dilution of mesogen concentration in the network [41]. Thus, dodecylamine was chosen as a chain extender because it resulted in the lowest *T*_NI_ for both DA2 and DA3 compared to other n‐alkyl primary amines that were tested (butylamine, hexylamine, and decylamine) (Figure S4b). *T*_NI_ is also affected by the degree of crosslinking. By increasing the ratio of maleimide to furan, which should increase the crosslink density of the LCEs, the *T*_NI_ for both DA2 and DA3 decreases (Figure 2a). The crosslinker (BMI) is a relatively high‐molecular‐weight molecule. As BMI content increases, the mesogen content in the network decreases. We also note that the *T*_rDA_ is somewhat higher than *T*_NI_ for both systems; as such, some crosslinks likely form in the isotropic phase during cooling or solvent evaporation. Crosslinking in the isotropic phase is known to reduce *T*_NI_ [13]. In this system, where crosslinks form on cooling in both the isotropic and nematic phases, the relative importance of this effect is not clear. The ratio of maleimide to furan that was chosen for the remaining experiments for both systems was 8.1:10. This ratio resulted in a relatively low *T*_NI_ while ensuring the maleimide is the limiting reagent.

DA2 and DA3 are comparable materials in many respects, differing primarily in the temperatures needed to dissociate the network. DA2 and DA3 exhibit three thermal transitions (Figures 2b,c). The lowest temperature transition for both DA2 and DA3 is a second‐order transition attributed to the glass transition. Glass transition temperatures are 29.2°C ± 1.4°C and 33.7°C ± 1.2°C for DA2 and DA3, respectively. We note that these glass transition temperatures correspond to equilibrium values obtained after complete dissociation of the DA bonds (after the first heating‐cooling cycle) and subsequent annealing of the samples at room temperature for 24 h. Room‐temperature annealing of DA2 and DA3 is essential, as immediately after complete dissociation of the DA bond network, the glass transition temperatures (*T*_g_) decrease to 0.3°C ± 1.6°C and 24.4°C ± 0.9°C for DA2 and DA3, respectively (Figure S5). The equilibrium *T*_g_ is attained after 24 h.

The intermediate temperature transition is an endothermic transition attributed to *T*_NI_. *T*_NI_ for DA2 and DA3 are 73.0°C ± 2.7°C, and 75.5°C ± 2.0°C, respectively. We note that the *T*_NI_ was measured with the same procedure that was followed for the *T*_g_. The *T*_NI_ of DA2 is not clearly visible after complete dissociation of the DA bonds and subsequent annealing of the samples at room temperature for 24 h, likely due to the overlapping dissociation of the DA network. However, after another heating cycle, the DSC for DA2 indicates a clear endothermic peak at 70.5°C ± 2.3°C (Figure S4c). The highest temperature transition is a broad endothermic peak that is attributed to the dissociation of DA bonds. The *T*_rDA_ is defined as the peak of that endothermic transition. The *T*_rDA_ for DA3 is 138.7°C ± 1.1°C, and for DA2 is 103.4°C ± 1.1°C. The nominal gaps between the *T*_NI_ and *T*_rDA_ are 30.4°C ± 1.6°C and 63.2°C ± 0.9°C for DA2 and DA3, respectively (Figure S4b). We highlight that *T*_rDA_ and *T*_NI_ are broad transitions for both DA2 and DA3; the nominal separation may not lead to complete separation of the two transitions during actuation. This nominal temperature separation is a design factor for the reversible actuation of the networks; however, it does not solely define the practical actuation window over which the actuator performs reversibly.

### 2.3 Actuation Properties of DA2 and DA3

**FIGURE 3:**
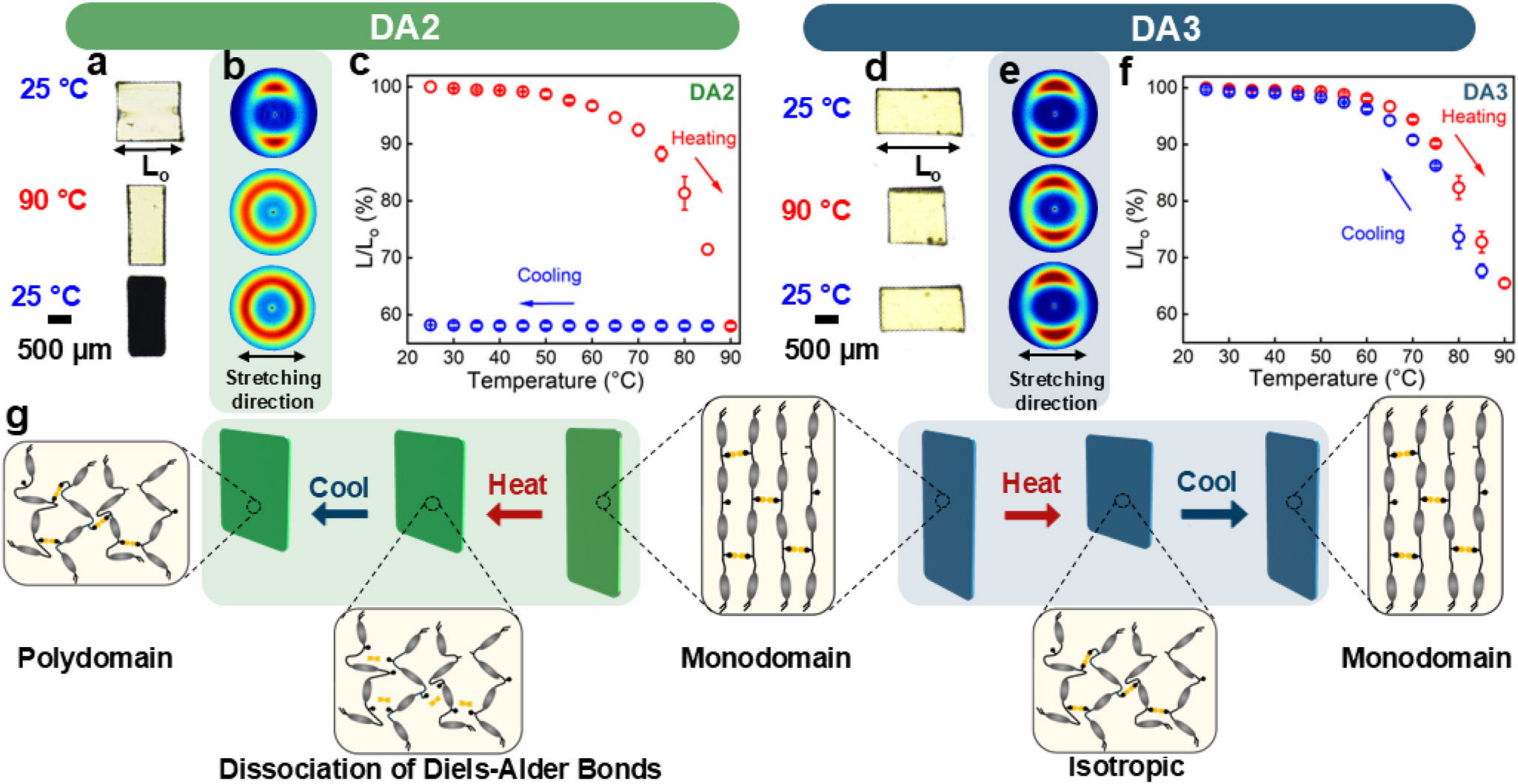
Shape changes of DA2 and DA3 in response to temperature. (a) Shape changes of DA2 at 25°C, 90°C, and 25°C during the first heating‐cooling cycle measured using brightfield unpolarized microscopy. (b) WAXS patterns of DA2 at 25°C, 90°C, and 25°C during the first heating‐cooling cycle. (c) Actuation strain of DA2 during the first heating‐cooling cycle from 25°C to 90°C. (d) Shape changes of DA3 at 25°C, 90°C, and 25°C during the first heating‐cooling cycle measured using brightfield unpolarized microscopy. (e) WAXS patterns of DA3 at 25°C, 90°C, and 25°C during the first heating‐cooling cycle. (f) Actuation strain of DA3 during the first heating‐cooling cycle from 25°C to 90°C. (g) Schematic of shape changes and phase transitions during heating‐cooling cycles of DA2 and DA3. Each test was repeated 3 times. Each data point represents the mean (*n* = 3), and the error bars represent the standard deviation.

It is well established in the literature that DA bonds formed with 3‐substituted furan are stronger than those with 2‐substituted furan, which, in non‐LCE networks, has been shown to translate into superior mechanical properties [44]. For an LCE actuator, it is crucial that the network does not dissociate to a degree that compromises the reversibility of actuation. Although the separation between *T*_NI_ and *T*_rDA_ is an important factor for enabling reversible actuation in DA2 and DA3, the nominal temperature gap alone does not fully define the practical actuation window within which these networks perform reversibly. Thus, solvent‐cast films of DA2 and DA3 samples can be programmed via mechanical alignment by simply stretching on a tensile tester at temperatures near *T*_rDA_, followed by cooling to room temperature (Figure 1b). After programming, the actuators are preheated at 80°C (which is above the *T*_NI_) to release residual stress, which would otherwise cause a partially irreversible response during the first cycle. Before actuation, homogeneous molecular alignment was confirmed in the DA2 and DA3 films by the presence of birefringence when observed between crossed polarizers (Figure S6). DA2 contracted upon heating from 25°C to 90°C without any external load but did not recover its original shape upon cooling back to 25°C, indicating an irreversible shape change under these conditions (Figure 3a). Heating to 90°C caused the sample to become nearly optically isotropic, indicating the transition from monodomain nematic to isotropic phase. On cooling, a transition from isotropic to polydomain nematic morphology is demonstrated by strong light scattering in the sample at 25°C (Figure 3a, Figure S7a). Upon heating above the *T*_NI_, the initial anisotropic “eyebrow” WAXS scattering pattern of the aligned monodomain nematic DA2 changed to a fully circular (isotropic) pattern, indicating the nematic‐to‐isotropic phase transition has occurred. After cooling, the pattern did not revert, indicating permanent loss of alignment upon cooling back to the nematic phase, which is consistent with a transition to a polydomain nematic state (Figure 3b). The order parameter decreased from 0.313 to −0.003 on heating and remained almost the same, −0.026, on cooling (Figure S8a,b,c). The small, apparent negative order parameter may arise from small forces on the sample during cooling (negative values are associated with a 90‐degree phase shift relative to the original alignment axis). During this process, the normalized length (*L*/*L*_o_) versus temperature revealed contraction of 42.0% ± 0.5% along the alignment direction on heating, but this deformation was irreversible (Figure 3c). We hypothesize that the monodomain nematic DA2 loses its order during the first heating cycle due to extensive dissociation of dynamic crosslinks (Diels–Alder bonds) at 90°C (Figure 3g).

The actuation performance of DA3 without a load showed reversible contraction upon heating from 25°C to 90°C and expansion upon cooling back to 25°C, indicating the reversible shape change of DA3 under these conditions (Figure 3d). The homogeneous molecular alignment of DA3 before heating was confirmed by the presence of birefringence (Figure S7b). After heating to 90°C, the sample contracted and remained somewhat birefringent, signifying a nematic‐to‐paranematic phase transition. Upon cooling, DA3 restored its shape and initial molecular alignment, as evidenced by the return to its initial length and birefringence. Heating to the nematic‐to‐paranematic phase transition temperature of DA3 results in a decrease in the order parameter from 0.285 to 0.139 (Figure 3e,S8d,e,f). Upon cooling, the order parameter recovered to 0.24, confirming reversible actuation. DA3 actuates reversibly with 34.5% ± 0.7% strain without substantial thermal hysteresis (Figure 3f). Upon heating from 25°C to 80°C, 90°C, and 100°C, the maximum temperature at which DA3 exhibits reversible actuation, the order parameter does not reach zero, and the diffraction pattern does not become fully circular, confirming that the transition is nematic‐to‐paranematic rather than nematic‐to‐isotropic (Figure S9). The reversible actuation of DA3 suggests that the gap between *T*_NI_ and *T*_rDA_ is sufficient to maintain alignment (Figure 3g).

We note that DA2 and DA3 oligomers had slightly different molecular weights at the same feed ratio of monomers. The number‐average molecular weight of the oligomer with the 2‐substituted furan was 7803 ± 62 g mol^−1^ (≈10 repeat units). The number‐average molecular weight of the oligomer with the 3‐substituted furan was 9935 ± 101 g mol^−1^ (≈13 repeat units) (Figure S10). Matching the molecular weight of the DA3 oligomer (DA3_matched_) to that of the DA2 oligomer was achieved by increasing the acrylate‐to‐amine ratio to 1.25:1, resulting in a DA3 oligomer molecular weight of 7901 ± 98 g mol^−^^1^ (≈10 repeat units) (Figure S11a,b). From ^1^H NMR spectra, the degree of polymerization was calculated from the ratio of the integrated peaks corresponding to the two furan protons of the X‐substituted furan (b protons) and the three methyl protons of the dodecylamine chain end (c protons) relative to the four acrylate protons (a protons), according to DP = (b/2 + c/3)/a/4 (Figure S3a–c). The average degree of polymerization was 8.5 for the polymeric precursor with the 2‐substituted furan, and the degree of polymerization for the polymeric precursor with the 3‐substituted furan was 11.6. Similarly, for DA3_matched_, ^1^H NMR analysis gave an average degree of polymerization of 7.6 with a furan‐to‐dodecylamine ratio of 3.1 (Figure S3c,S10). The values of degree of polymerization by ^1^H NMR are consistent with the degree of polymerization estimated from gel permeation chromatography. DA3_matched_ with the matched precursor molecular weight also showed a T_NI_ at 77.0°C ± 3.2°C (Figure S11c), which is slightly higher than that of DA3. DA3_matched_ exhibited reversible actuation for at least five heating‐cooling cycles (Figure S11d); however, it showed a lower actuation stress of 74.2 ± 13.3 kPa compared to DA3 (Figure S11e). DA3_matched_ has a slightly lower T_g_ as compared to DA3 (Figure S11f). This suggests that differences in chain entanglement between DA2 and DA3 are not responsible for the differences in actuation behavior.

**FIGURE 4:**
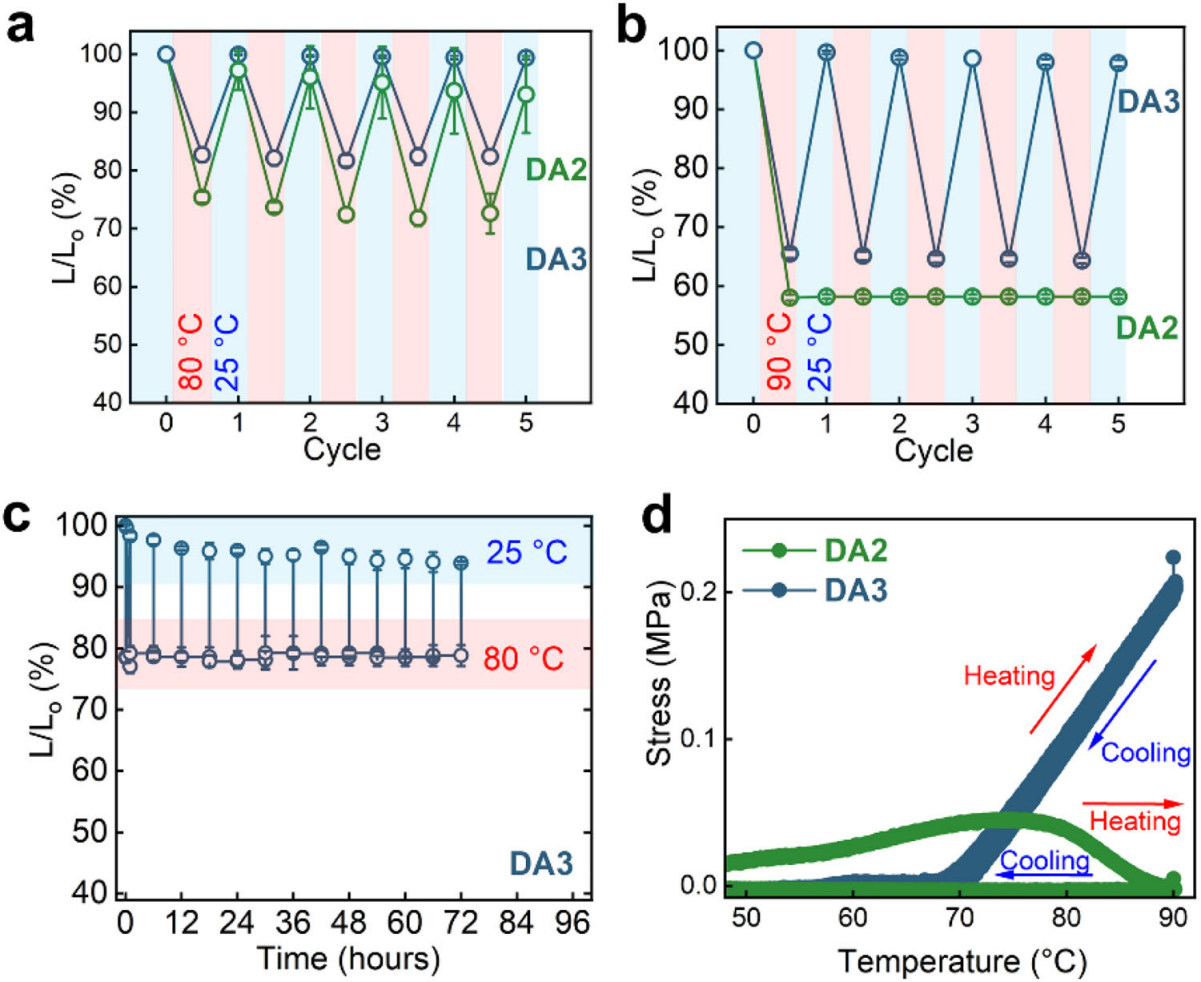
(a) Actuation strain of DA2 and DA3 from 25°C to 80°C for 5 heating‐cooling cycles. (b) Actuation strain of DA2 and DA3 from 25°C to 90°C for 5 heating‐cooling cycles. c) Actuation strain of DA3 during storage at 80°C over 72 h, with cooling and length measurement performed every 6 h. (d) Representative actuation stress of DA2 and DA3 from 50°C to 90°C. Each test was repeated 3 times. Each data point represents the mean (*n* = 3), and the error bars represent the standard deviation.

Reversible actuation and irreversible loss of alignment can be observed in both DA2 and DA3, depending on the conditions. DA2 demonstrated fully reversible actuation over 5 heating‐cooling cycles with 8.1% ± 0.6% strain when the maximum temperature was limited to 70°C (Figure S12). Increasing the maximum temperature to 80°C increased the change in length to 26.8% ± 1.4%, but loss of actuation stability occurred during repeated cycling, likely due to partial DA bond dissociation (Figure 4a). At 90°C maximum temperature, DA2 failed to recover from the first actuation cycle, indicating that a significant fraction of DA bonds was broken (Figure 4b). In contrast, DA3 maintained completely reversible actuation throughout 5 cycles at maximum temperatures up to 100°C (Figure S13a). The actuation strain increased from 9.4% ± 0.3% to 17.7% ± 0.4% and 35.2% ± 0.5% at maximum temperatures of 70°C, 80°C, and 90°C, respectively (Figures 4a,b, S12). At 100°C maximum temperature, DA3 maintained reversible actuation with 37.5% ± 0.2% strain (Figure S13a). However, at 110°C, actuation performance degraded through actuation cycles with strain decreasing from 40.2% ± 0.2% in the first cycle to 13.4% ± 7.9% by the fifth cycle (Figure S13b). At 120°C, DA3 failed to recover from the first actuation cycle, indicating a critical dissociation of the network (Figure S13c).

The actuation performance of DA3 was further evaluated by exposing the material to an extended period of 72 h at 80°C. Initially, DA3 was tested over two heating‐cooling cycles to demonstrate its reversibility. After cycle 2, DA3 exhibits 21.4% ± 0.0% reversible strain. Subsequently, the material was continuously exposed to 80°C for 72 h with cooling to room temperature every 6 h to measure actuation. After this accelerated aging experiment, DA3 exhibits 21.4% ± 0.6% reversible actuation (Figure 4c). At the conclusion of this test, DA3 samples were exposed to 110°C for 1 h, during which they contracted by 34.1% ± 0.8%. However, upon cooling, it failed completely to return to its initial shape, indicating the expected irreversible loss of alignment (Figure S14). On the other hand, DA2 was initially tested over two heating‐cooling cycles to demonstrate its reversibility. Subsequently, it was exposed to 80°C for 1 h, and upon cooling, the shape change was completely irreversible (Figure S15).

To perform work on the environment, the actuator must operate against a load. Thus, blocking stress is another crucial parameter to evaluate the actuation performance of LCEs. DA3 generates 213.7 ± 20.1 kPa actuation stress on heating to 90°C, while DA2 exhibits significantly lower stress values at equivalent temperatures, and actuation stress drops to zero above 80°C (Figure 4d). DA2 maintains effective crosslinks only up to 80°C, which is 30.4°C ± 1.6°C below the nominal *T*_rDA_ of 103.4°C ± 1.1°C, demonstrating the effect of the broad DA bond dissociation transition. DA3 can operate stably up to 100°C, which enables a larger actuation stress given the broad nature of the phase transition behavior of this material.

**FIGURE 5:**
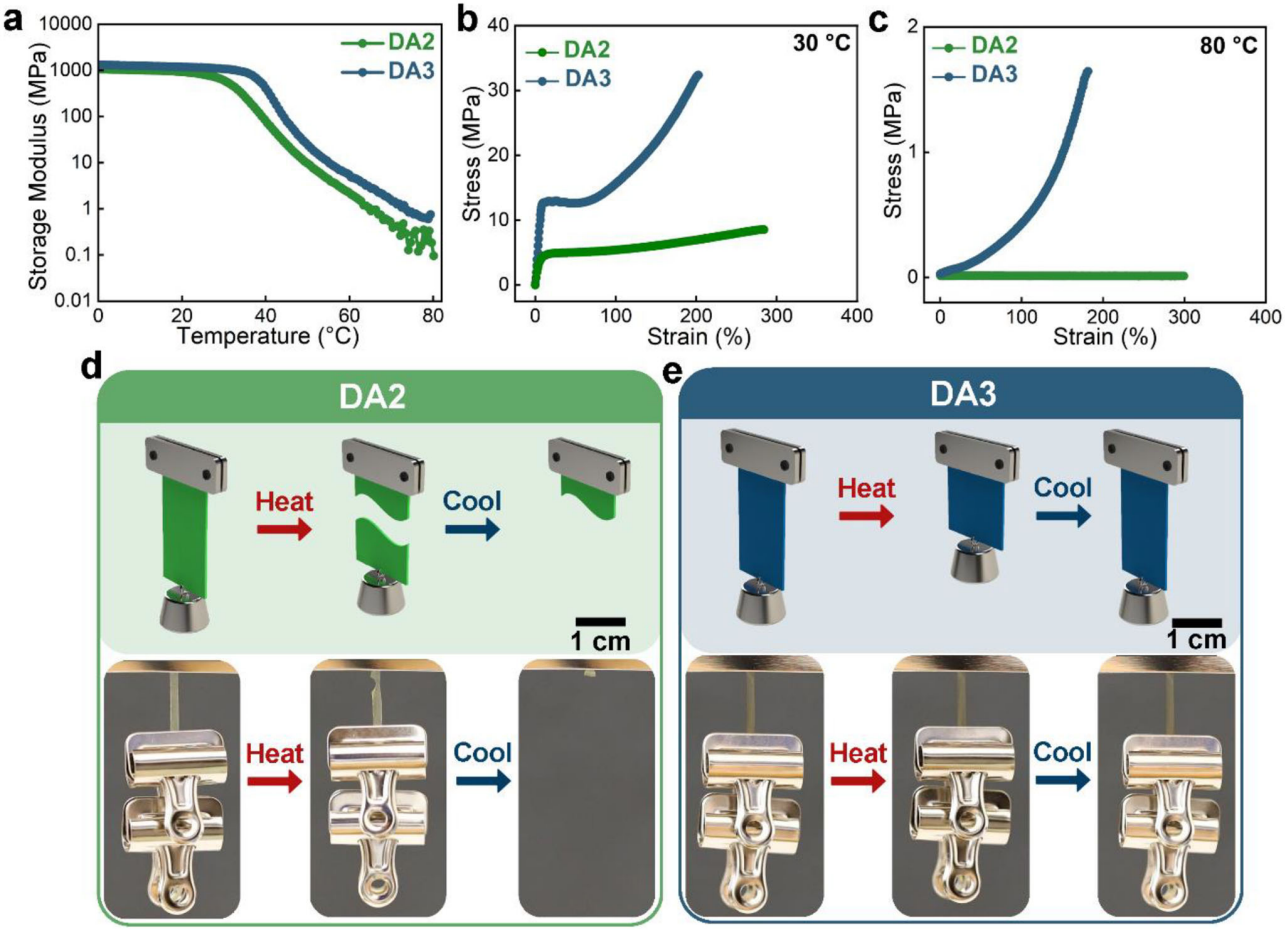
(a) Representative storage modulus of DA2 and DA3 from 0°C to 80°C. (b) Representative stress–strain of DA2 and DA3 at 30°C. (c) Representative stress–strain of DA2 and DA3 at 80°C. (d) Thermal stability of DA2 under load at 90°C. (e) Thermal stability of DA3 under load at 90°C. Each test was repeated 3 times.

The transition temperatures affect the mechanical properties of DA2 and DA3. Storage modulus as a function of temperature of unaligned materials shows that DA2 and DA3 exhibit similar behavior at ambient temperatures. However, above 70°C, DA2 shows lower values of storage modulus (Figure 5a). At 30°C, DA2 and DA3 have comparable modulus values, with DA2 having lower stress at break than DA3 (Figure 5b). With increasing temperature, the stress at break for both DA2 and DA3 drops dramatically. At 80°C, which is above the *T*_NI_, DA2 flows upon stress, whereas DA3 has a 1.6 ± 0.0 MPa stress at break (Figure 5c), comparable to that of some permanently crosslinked LCEs measured at 10°C above *T*_NI_ [12]. The superior thermal stability of DA3 compared to DA2 is clearly demonstrated when lifting a load. After aligning DA2 and DA3, weights were attached to each sample to apply a stress of 283.4 kPa. DA2 and DA3 were subjected to heating and cooling cycles under load (Figure S16, Movie S1). DA2 breaks under load after the third heating cycle at 90°C (Figure 5d, S16a, Movie S1), whereas DA3 is capable of performing actuation of at least 5 cycles without breaking (Figure 5e, S16b, Movie S1).

### 2.4 Reprogrammability and Recyclability of DA3

**FIGURE 6:**
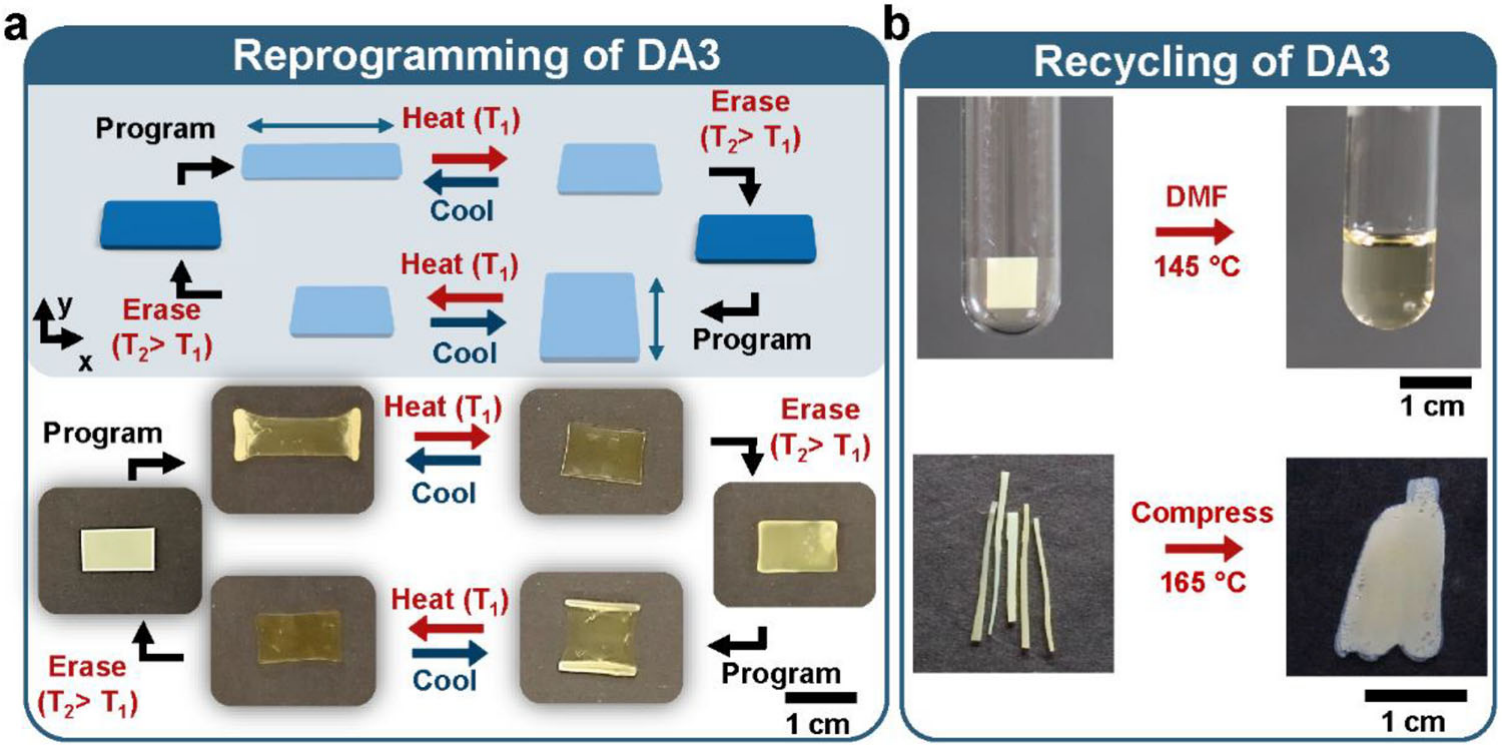
(a) Schematic (top) and experiment (bottom) of reprogramming of DA3 on the x‐ and y‐axis, (b) recycling of DA3 in DMF, and under compression.

The ability of LCEs with DA bonds to be reprogrammed and reprocessed is a key advantage over LCEs with permanent covalent crosslinks. The reprocessability of LCEs with DA bonds has previously been reported for DA2‐type bonds [30, 37]. Accordingly, the reprogramming and reprocessing capabilities of DA3 were evaluated. To erase programmed alignment of DA3 monodomain and restore the polydomain state, the aligned film was heated to 135°C (*T*_2_) to induce dissociation of the network (Figure 6a). The alignment process can then be repeated to fabricate an actuator with a distinct shape change. Dissociation of the network enables facile dissolution and melt processing [17]. Polydomain DA3 dissolves in DMF at 145°C (Figure 6b). Additionally, DA3 fragments were compressed at 165°C. After 4 min, the pieces flowed together, showing the capability of the DA3 to be melt‐processed (Figure 6b). DA2 demonstrated reprocessability through both dissolution and compression methods at 120°C (Figure S17). These results also indicate a lack of undesired permanent covalent crosslinks, such as those formed from acrylate homopolymerization.

### 2.5 Dynamic Diffraction Grating of DA3 for Evaluating Actuator Performance

**FIGURE 7:**
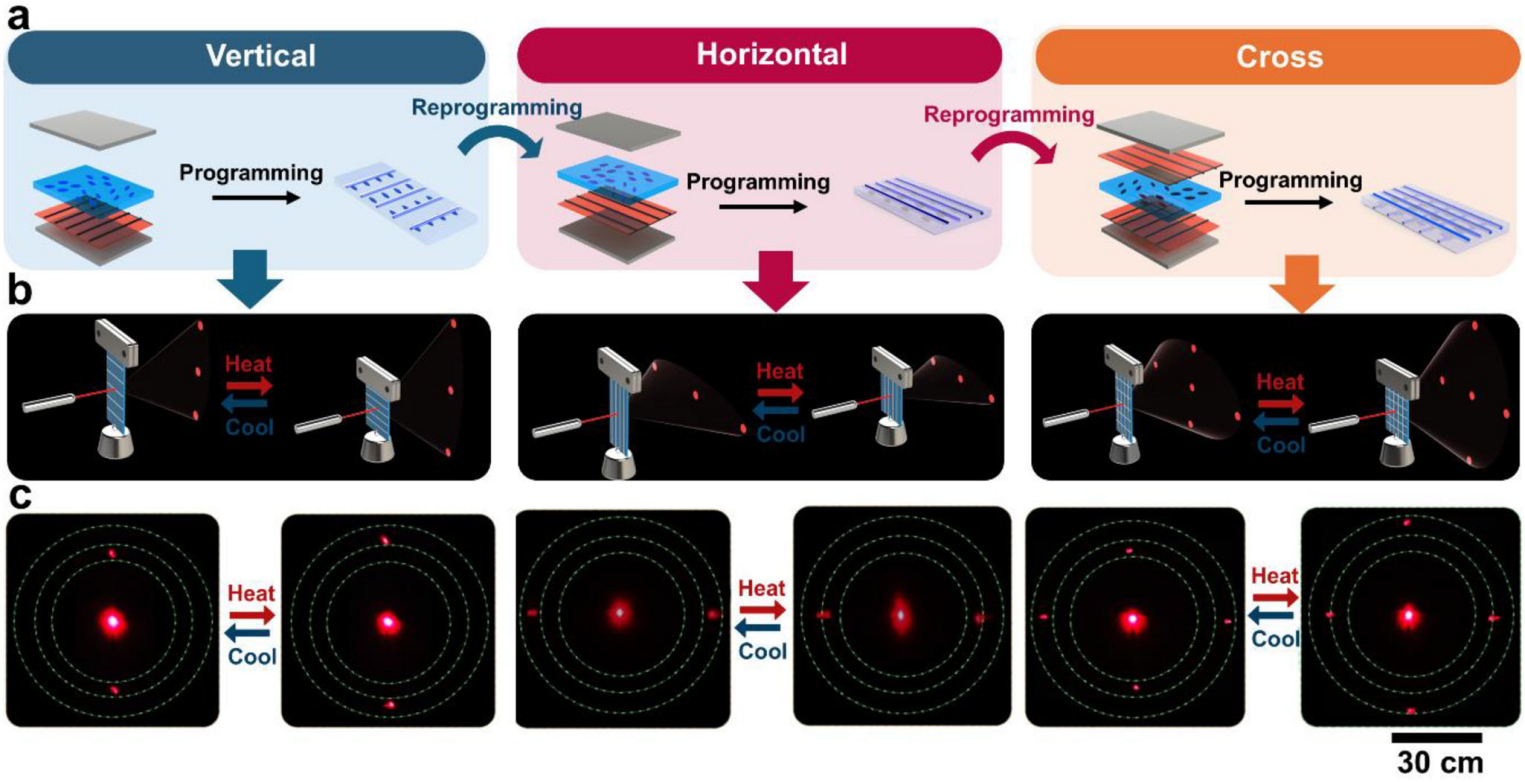
(a) Schematic of the fabrication of the DA3 dynamic diffraction grating actuator in vertical, horizontal, and cross‐aligned orientations with respect to the molecular alignment direction, and laser diffraction changes of the actuator under constant load during heating‐cooling cycles, (b) diffraction pattern changes during thermal cycling of the DA3 dynamic diffraction grating. Green circles highlight changes in spot positions during actuation.

The ability to monitor shape changes in soft actuators is key to integrating them into engineered systems [7, 50, 51]. LCEs have been micropatterned to create dynamic diffraction gratings, which could be used to monitor actuation or serve as dynamic optical elements [52, 53, 54, 55, 56]. The incorporation of dynamic bonds into LCEs improves processability and facilitates the imprinting of micropatterns. First, a micropatterned polydomain DA3 was prepared by hot‐pressing the film onto a commercially available diffraction grating (Figure 7a, Figure S18a). Then the material was aligned using the previously described method (Figure 7a,S18b). The aligned DA3 dynamic diffraction grating exhibits reversible actuation from 25°C to 90°C. With the grating line perpendicular to the alignment, the spacing decreased from 3.3 to 2.3 µm during thermal actuation (Figure S19). The same procedures applied to DA2 resulted in a complete loss of grating structure after a single actuation cycle. To evaluate the performance of the DA3 dynamic diffraction grating actuator under load, a clip was attached to the actuator to apply a stress of 39.1 kPa, and a laser was directed onto the sample. When the sample was heated to 90°C, the sample contracted and the distance between the diffraction spots expanded because the distance between the lines shortened (Figures 7b,c, Movie S2). Upon cooling, the sample recovered its initial shape, and the diffraction pattern returned to its initial position. The same DA3 sample was then micropatterned and reprogrammed by aligning along the grating lines (Figure 7a). Upon heating from 25°C to 90°C, the distance between the diffraction spots decreased because of the increasing distance between the lines of the diffraction grating. Finally, the same DA3 film was micropatterned by sandwiching DA3 between two grating films positioned perpendicular to each other and aligned. The diffraction pattern consisted of four dots. During heating‐cooling cycles, the patterns followed the changes described for the previous two DA3 dynamic gratings. For the DA3 diffraction grating actuator in cross‐aligned orientation, the diffraction angle of the laser was calculated using Bragg's equation, based on the geometry of the diffraction setup, measured grating spacing at room temperature, and measured grating spacing at 90°C (Figure S19). The analysis showed that the difference between the theoretical and experimental values was 3.2% ± 7.4%, confirming good agreement between the two (Figure S20, Movie S2). Thus, the diffraction spots from the laser could be used to monitor the performance of the actuator in a noncontact mode, which may be useful in certain robotic applications.

## 3 Conclusions

Reprocessable and recyclable LCE actuators were prepared with DA bonds that differ in regiochemistry. 3‐substituted furans lead to a more thermally stable network as compared to crosslinks with 2‐substituted furans. The length of the n‐alkyl amine chain extenders and the degree of crosslinking tuned the actuation temperature, while the furan substitution controlled the dissociation temperature of the DA bonds. LCEs made with more thermally stable DA bonds have a wider temperature window for reversible actuation, while preserving the reprogrammability and reprocessability. Leveraging reprocessability of DA3, a reprogrammable actuator micropatterned with a diffraction grating can be fabricated, which enables noncontact measurement of actuator shape. The combination of reprogrammability and thermal stability may enable the use of these LCE actuators in applications including soft robotics.

## Conflicts of Interest

The authors declare no conflicts of interest.

## Supporting information




**Supporting File 1**: smll74705‐sup‐0001‐SuppMat.docx.

**Supporting File 2**: smll74705‐sup‐0002‐MovieS1.mp4.

**Supporting File 3**: smll74705‐sup‐0003‐MovieS2.mp4.

## Data Availability

The data that support the findings of this study are available from the corresponding author upon reasonable request.
