# Supplementary material for: Enhanced Actuation Stability of Liquid Crystal Elastomer Actuators With Diels–Alder Dynamic Bonds
Source: Small. 2026 Aug 2;22(53):e74705. doi: 10.1002/smll.74705 (PMC13592405; doi:10.1002/smll.74705)
Supplement: Supplementary file 1 [file SMLL-22-e74705-s001.docx]

Supporting Information

**Enhanced Actuation Stability of Liquid Crystal Elastomer Actuators with Diels-Alder Dynamic Bonds**

Konstantinos Stamatis ^a^, Yoo Jin Lee ^b^, Asaf Dana ^a,b^, Manivannan Sivaperuman Kalairaj ^b^, Caleb A. Crusco ^a^, Jared A. Gibson ^c, d^, Sasha M. George ^a^, Svetlana A. Sukhishvili ^a*^, Taylor H. Ware ^b, a *^

Table of contents

[Experimental procedure	1](#_Toc231818035)

[Materials	1](#_Toc231818036)

[Synthesis of the precursor polymer for liquid crystal elastomers (LCEs) with Diels-Alder (DA) bonds using different chain extenders with ratio acrylate-to-amine molar ratio of 1.1:1	2](#_Toc231818037)

[Synthesis of the precursor polymer for DA2 and DA3 with ratio acrylate-to-amine molar ratio of 1.25:1	2](#_Toc231818038)

[Crosslinking method of LCEs with DA bonds	2](#_Toc231818039)

[Alignment method of LCEs with DA bonds	2](#_Toc231818040)

[Structural characterization and molecular weight determination of precursor polymers with X-Substituted furan	3](#_Toc231818041)

[Thermal characterization of LCEs with DA bonds	3](#_Toc231818042)

[Mechanical characterization of DA2 and DA3	3](#_Toc231818043)

[Optical properties and actuation performance of LCEs with DA bonds	3](#_Toc231818044)

[Wide-Angle X-ray Scattering (WAXS) OF DA2 and DA3	4](#_Toc231818045)

[Thermal stability of DA2 and DA3 under load	4](#_Toc231818046)

[Reprogrammability of DA3	4](#_Toc231818047)

[Recyclability of DA2 and DA3	4](#_Toc231818048)

[Fabrication of dynamic diffraction grating	5](#_Toc231818049)

[Actuation of dynamic LCE diffraction grating	5](#_Toc231818050)

[Results and discussion	6](#_Toc231818051)

# **Experimental procedure**

**Materials**

Liquid-crystal monomer 1,4-Bis-[4-(6-acryloyloxypropyloxy)benzoyloxy]-2-methylbenzene (RM82, purity: 97 %) was purchased from Henan Daken Chemical, furan-3-ylmethanamine (3-substituted furan, purity 95 %) was purchased from Angene Chemical. Furan-2-ylmethanamine (2-substituted furan, purity 98 %), dodecan-1-amine (dodecylamine, purity 98 %), and dichloromethane (CH_2_Cl_2_, purity 99.8 %) were purchased from Acros Organics. 1,1′-(Methylenedi-4,1-phenylene)bismaleimide (BMI, purity 95 %) was purchased from Sigma-Aldrich. Dimethylformamide (DMF, purity 99.8 %) was purchased from Sigma-Aldrich. Silicone oil (density 0.963) was purchased from Thermo Scientific. A diffraction grating sheet with a single linear axis of 500 lines/mm was purchased from Bartovation. A red laser pointer (class IIIA, <5mW, 650nm) was purchased from Hart Tools. All chemicals used in this study were used as received without further purification. Images were captured using a Canon EOS Rebel T7i DSLR camera equipped with a 100 mm external lens. Temperature changes were captured using FLIR A400 image-streaming thermal camera.

Synthesis of the precursor polymer for liquid crystal elastomers (LCEs) with Diels-Alder (DA) bonds using different chain extenders with ratio acrylate-to-amine molar ratio of 1.1:1

For the synthesis of the precursor polymer with furan-x-ylmethanamine (where x = 2 or 3), a mixture of RM82 (500 mg, 0.74 mmol), furan-x-ylmethanamine (50.7 mg, 0.46 mmol), and dodecan-1-amine (24.1 mg, 0.11 mmol) was mixed in a 20 mL vial, heated with a heat gun around 20 seconds until the opaque mixture became transparent, and vortexed for 2 min to ensure homogeneous mixing. The vial was then placed in an oil bath and stirred at 75 °C for 12 hours. The same procedure was used for synthesizing the precursor polymers with the other aliphatic primary amines (butylamine, hexylamine, and decylamine).

Synthesis of the precursor polymer for DA2 and DA3 with ratio acrylate-to-amine molar ratio of 1.25:1

For the synthesis of the precursor polymer with furan-x-ylmethanamine, a mixture of RM82 (500 mg, 0.74 mmol), furan-x-ylmethanamine (44.6 mg, 0.52 mmol), and dodecan-1-amine (21.2 mg, 0.13 mmol) was mixed in a 20 mL vial, heated with a heat gun around 20 seconds until the opaque mixture became transparent, and vortexed for 2 min to ensure homogeneous mixing. The vial was then placed in an oil bath and stirred at 75 °C for 12 hours.

Crosslinking method of LCEs with DA bonds

The precursor polymer (200 mg) was dissolved in 5 mL of CH_2_Cl_2_. 1,1′-(Methylenedi-4,1-phenylene) bismaleimide (BMI) (42.3 mg, 0.11 mmol) was dissolved in the precursor polymer solution to achieve a ratio of maleimide to furan of 8.1:10 unless otherwise noted, and the mixture was stirred for 2 hours. After stirring, the solution was poured into a rectangular Teflon mold with dimensions 85 mm × 27 mm × 2.7 mm and allowed to air-dry at room temperature until the solvent had completely evaporated. The thickness of the mold is 2.7 mm. After the evaporation of the solvent, the typical thickness of the polydomain is 2.2 mm. The thickness of each tested sample was determined by averaging three measurements taken at different points across the sample. The crosslinked sample was then annealed for 12 hours at 75 °C to form the network. After annealing, the sample was kept at room temperature for 24 hours to allow full network formation. The typical thickness of the monodomain is 0.19 mm. The crosslinking method was the same for all the precursors. The same procedure was followed in order to achieve a ratio of maleimide to furan of 2.5:10, 5:10, 7.5:10, and 10:10, varying the amount of BMI.

Alignment method of LCEs with DA bonds

A dynamic mechanical analyzer (DMA) (TA Instruments, RSA-G2) was used to align the LCEs with DA bonds. Initially, rectangular strips of LCEs with DA bonds having furan-2-ylmethanamine with dimensions of approximately 7.8 mm × 2.8 mm × 0.22 mm were preheated to 90 °C for 5 minutes and subjected to 20% strain at a constant rate of 0.04 mm/s at the same temperature. The load is then fixed during cooling from 90 to 25 °C, with a temperature ramp rate of 10.0 °C /min. Rectangular strips of LCEs with DA bonds having furan-3-ylmethanamine with dimensions of approximately 8 mm × 2.5 mm × 0.2 mm were preheated to 135°C for 5 minutes and subjected to 20% strain at a constant rate of 0.04 mm/s at the same temperature. The load is then fixed during cooling from 135 to 25 °C, with a temperature ramp rate of 10.0 °C /min. After programming, each actuator was preheated at 80 °C (which is above the T_NI_) for 2 min to release residual stress, which would otherwise cause an irreversible response during the first cycle.

Structural characterization and molecular weight determination of precursor polymers with X-Substituted furan

Gel Permeation Chromatography was conducted to determine the molecular weight of the oligomers. The oligomers were dissolved in dimethylformamide (DMF) at a concentration of 3 mg/mL for 24 hours at room temperature. The prepared solutions were then loaded into an Agilent 1260 Infinity II HPLC GPC system with a flow rate of 0.1 mL/min at room temperature. The molecular weight characterization was repeated 3 times (*n* = 3).

The furan-to-dodecylamine ratio and degree of polymerization (DP) were characterized by ^1^H NMR spectroscopy using a Bruker Avance Neo 400 MHz spectrometer equipped with an Ascend magnet and a 5 mm broadband iProbe. All spectra were recorded in CDCl_3_ as the solvent. The ¹H NMR measurements for each precursor polymer were repeated 3 times (n = 3).

Thermal characterization of LCEs with DA bonds

Differential scanning calorimetry (DSC) was conducted to determine the T_g_, T_NI_, and T_rDA_. Approximately 5 mg of each sample was weighed and sealed in Tzero aluminum pans and lids (TA Instruments). The prepared samples were then loaded into the DSC instrument (TA Instruments DSC 2500). The temperature range was from -10 to 160 °C with a temperature ramp rate of 10 °C/min under an N_2_ gas flow of 50 mL/min. We note that these glass transition temperatures correspond to equilibrium values obtained after complete dissociation of the DA bonds and subsequent annealing of the samples at room temperature for 24 h. The thermal analysis was repeated 3 times (*n* = 3).

Mechanical characterization of DA2 and DA3

Dynamic mechanical analyzer (DMA) (TA Instruments, RSA-G2) was used for the mechanical characterization of the materials. For quasi-static tensile testing, polydomain LCEs were prepared. Rectangular strips of LCE with DA bonds with dimensions of approximately 4.5 mm × 1.4 mm × 0.2 mm were loaded and stretched at a constant linear rate of 0.04 mm/s. Testing was performed at various constant temperatures. Before each experiment, samples were preheated for 5 minutes to the testing temperature to ensure thermal equilibrium.

For dynamic mechanical analysis, polydomain samples having dimensions of approximately 10 mm × 1.1 mm × 0.2 mm were loaded and tested in tensile mode at 0.2 % strain at 1 Hz while being heated from -10 to 80 °C at a rate of 3 °C /min.

For cyclic blocking stress measurements, the temperature was ramped from 25 to 90 °C and back to 25 °C twice at a rate of 2.0 °C /min. Uniaxially aligned sample dimensions were approximately 3 mm × 0.7 mm × 0.2 mm.

All tests were repeated three times for each composition (*n* = 3).

Optical properties and actuation performance of LCEs with DA bonds

Polarized Optical Microscopy (POM) (Nikon Eclipse LV100 POL) was used to determine the optical and actuation properties of the aligned LCEs with DA bonds. Samples were cut with dimensions of approximately 1 mm × 0.7 mm × 0.2 mm and placed on glass slides with silicone oil to prevent adhesion. A thermal stage (Linkam LTS420 thermal stage) was used to heat and cool the sample. Five heating-cooling cycles were conducted from 25 °C to various maximum temperatures, increasing in 5 °C increments, to evaluate the shape change. At the highest temperature, the sample was allowed to remain for 5 min.

The normalized length along the nematic director was measured using the following equation:

$$\frac{L}{L_{o}}(\%)$$

where L_o_ is the initial length, and L is the final length.

The actuation performance measurements were repeated 3 times for all conditions (n = 3).

Wide-Angle X-ray Scattering (WAXS) OF DA2 and DA3

WAXS was collected using a Xeuss 3.0 system from Xenocs (Soft Matter Facility, Texas A&M University). Sample temperature was controlled using a Modular Force Stage (Linkam) connected to a T96-S controller (Linkam). The incident X-ray wavelength was λ=1.54189 Å. Full Debye-Scherrer rings were integrated over 300 s intervals on a DECTRIS Si 1M Eiger photon-counting area detector at different temperatures.

WAXS was employed to probe the order parameter and diffraction patterns of aligned LCEs with DA bonds throughout a heating-cooling cycle. Aligned DA2 and DA3 samples, measuring approximately 1.6 mm × 0.4 mm × 0.25 mm, were placed on a Modular Force Stage (Linkam) under zero applied force. WAXS data were collected by heating the sample from 25 °C to 80, 90, and 100 °C, followed by cooling to 90, 80, and 25 °C. Scattering patterns were recorded at each temperature after equilibrating for 5 minutes. The reversibility of the actuators was assessed by inspecting the obtained scattering patterns and calculating the corresponding order parameter.

Xenocs XSACT software and self-developed MATLAB-based scripts were used to analyze the stored images. The data analysis relies on image processing of the recorded scattering patterns. To reduce noise and artifacts from air scattering, the background intensity, taken under the exact same experimental conditions without the presence of the sample, was subtracted from the recorded patterns.

Thermal stability of DA2 and DA3 under load

Uniaxially aligned films with dimensions 21 mm × 2.7 mm × 0.2 mm were clamped in a clip secured to a horizontal stand. The alignment direction of the strip was oriented perpendicular to the ground. Two clips were attached to the bottom of the strip to provide a constant load. The strip was then heated with a heat gun to 90 °C approximately for 3 seconds, and the temperature was monitored using an IR camera.

Reprogrammability of DA3

A rectangular polydomain DA3 strip with dimensions 10 mm × 7 mm × 0.25 mm was cut such that alignment is along the x-axis (shorter dimension) using the previously described alignment method. The aligned sample was left at room temperature for 24 h to fully form the network. A petri dish with silicone oil was placed on a hot plate. The aligned sample was placed in the oil bath, and three heating-cooling cycles were performed from 25 to 90 °C. After actuation, the aligned sample was heated to 135 °C. The sample was then realigned along the y-axis (longer dimension) and actuated following the same procedure described previously.

Recyclability of DA2 and DA3

A rectangular polydomain DA2 strip (approximately 10 mm × 7 mm × 0.25 mm) was placed in a glass test tube containing about 2 mL of DMF and heated in an oil bath at 120 °C for 5 minutes, then removed and allowed to cool. Rectangular polydomain DA3 strips (approximately 10 mm × 7 mm × 0.25 mm) were each placed in separate glass test tubes containing about 2 mL of DMF. The tubes with DA3 were heated in an oil bath at 145 °C for 5 minutes, then removed and allowed to cool.

Four DA2 strips (approximately 13 mm × 1 mm × 0.25 mm) were heated and pressed between two glass slides at 120 °C for 2 minutes. Five DA3 strips (approximately 13 mm × 1 mm × 0.25 mm) were heated and pressed between two glass slides at 165 °C for 4 minutes. All the glass slides were coated with Rain-X.

Fabrication of dynamic diffraction grating

A commercially available single-axis diffraction grating sheet with 500 lines/mm was coated with Rain-X and placed on a glass slide, with the grating side facing away from the glass. The glass slide with the grating was placed on a hotplate and heated to 120 °C. A polydomain rectangular DA2 strip with dimensions 25 mm × 10 mm × 0.2 mm was placed on the glass slide with the grating and covered with another glass slide coated with Rain-X. The top slide was pressed down until material flow was observed. The grating-molded DA2 was removed from the hotplate and allowed to cool to room temperature. It was then separated from the commercial grating, and a strip with dimensions 19 mm × 4 mm × 0.1 mm was cut and aligned as previously described.

A commercially available single-axis diffraction grating sheet with 500 lines/mm was coated with Rain-X and placed on a glass slide, with the grating side facing away from the glass. The glass slide with the grating was placed on a hotplate and heated to 190 °C. A rectangular DA3 strip with dimensions 25 mm × 10 mm × 0.2 mm was placed on the glass slide with the grating and covered with another glass slide coated with Rain-X. The top slide was pressed down until material flow was observed. The grating-molded DA3 was removed from the hotplate and allowed to cool to room temperature. It was then separated from the commercial grating, and a strip with dimensions 20 mm × 5 mm × 0.1 mm was cut and aligned as previously described. The aligned sample was left at room temperature for 24 h to fully form the network. After the test, the aligned sample was heated to 135 °C in order to erase the diffraction grating and the programming. The sample was then micropatterned with the diffraction grating and realigned following the same procedure described previously.

Actuation of dynamic LCE diffraction grating

Actuation of the DA3 dynamic diffraction grating was carried out in a dark room to enhance visibility of the diffraction pattern. A rectangularly aligned DA3 strip with dimensions 18.1 mm × 4.3 mm × 0.07 mm was clamped in a clip secured to a horizontal stand. The alignment direction of the strip was oriented perpendicular to the ground. A clip was attached to the bottom of the strip to apply a constant stress of 39.1 kPa. A 650 nm laser was directed at the DA3 dynamic diffraction grating, producing a diffraction pattern on the wall. The strip was then heated with a heat gun to 90 °C approximately for 3 seconds, and its temperature was monitored using an IR camera. During the heating-cooling cycles, the distance between the diffraction spots and the grating was recorded using a Canon EOS Rebel T7i DSLR camera equipped with a 100 mm external lens.

Results and discussion

Figure S1. a) Synthesis of oligomeric polymer precursor furan-(2)-ylmethanamine. b) Synthesis of oligomeric polymer precursor furan-(3)-ylmethanamine.

Figure S2. a) Diels-Alder reaction for Liquid Crystal Elastomers with furan-(2)-ylmethanamine, b) Diels-Alder reaction for Liquid Crystal Elastomers with furan-(3)-ylmethanamine.

Figure S3. a) ^1^H NMR spectra of oligomeric polymer precursor with furan-(2)-ylmethanamine for DA2, b) ^1^H NMR spectra of oligomeric polymer precursor with furan-(3)-ylmethanamine for DA3, c) ^1^H NMR spectra of oligomeric polymer precursor with furan-(3)-ylmethanamine for DA3_matched_. Each test was repeated 3 times.

Figure S4. a) Nematic to isotropic transition temperature (T_NI_) and retro-Diels-Alder Dissociation temperature (T_rDA_) for DA2 and DA3 with different amines, b) Difference between the retro-Diels-Alder Dissociation temperature (T_rDA_) and nematic to isotropic transition temperature (T_NI_) for DA2 and DA3 with different amines. c) Representative DSC curve of the third heating cycle for DA2. Each test was repeated 3 times. Each data point represents the mean (n = 3), and the error bars represent the standard deviation.

Figure S5. Effect of DA bond formation on the glass transition temperature (T_g_) of DA2 and DA3 after complete DA bond dissociation. Each test was repeated 3 times. Each data point represents the mean (n = 3), and the error bars represent the standard deviation.

Figure S6. a) DA2 parallel to the analyzer (left) and rotated by 45 ° with respect to the analyzer (right). Arrows indicate the orientation of the analyzer and polarizer. b) DA3 parallel to the analyzer (left) and rotated by 45 ° with respect to the analyzer (right). Arrows indicate the orientation of the analyzer and polarizer.

Figure S7. a) Polarized optical microscopy images of DA2 at 25, 90, and 25 °C during the first heating-cooling cycle. b) Polarized optical microscopy images of DA3 at 25, 90, and 25 °C during the first heating-cooling cycle. The images were acquired using polarized optical microscopy. Each test was repeated 3 times. Arrows indicate the orientation of the analyzer and polarizer.

Figure S8. 1D β-phase azimuthal intensity versus angle extracted from the 2D WAXS patterns for DA2 at (a) 25 °C, (b) 90 °C, and (c) 25 °C after cooling, and again at (d) 25 °C, (e) 90 °C, and (f) 25 °C after cooling. These profiles form the basis for calculating the orientational order parameter.

Figure S9. a) WAXS patterns of DA3 at 25, 80, 90, 100, 90, 80, and 25 °C during the first heating-cooling cycle. b) Orientational order parameter of DA3 at 25, 80, 90, 100, 90, 80, and 25 °C during the first heating-cooling cycle.

Figure S10. a) Experimental vs theoretical molecular weight of DA2 and DA3. b) Experimental vs theoretical number of repeat units of DA2 and DA3. Each test was repeated 3 times. Each data point represents the mean (n = 3), and the error bars represent the standard deviation.

Figure S11. a) Experimental vs theoretical molecular weight of DA2 and DA3_matched_ (DA3 with acrylate-to-amine molar ratio of 1.25:1). b) GPC spectra of DA2, DA3_matched_ and DA3 with number-average molecular weights of 7803 ± 62 g mol^-1^ (≈10 repeat units), 7901 ± 98 g mol^-1^ (≈10 repeat units) and 9935 ± 101 g mol^-1^ (≈13 repeat units), respectively. c) Representative DSC curve of the second heating cycle for DA3_matched_. d) Actuation of DA2 and molecular weight-matched DA3 from 25 to 90 °C. e) Representative actuation stress of DA3_matched_ from 50 to 90 °C. f) Representative storage modulus of DA3_matched_ from 0 to 80 °C. Each test was repeated 3 times. Each data point represents the mean (n = 3), and the error bars represent the standard deviation.

Figure S12. Actuation strain of DA2 and DA3 from 25 to 70 °C for 5 heating cooling cycles.

Figure S13. Actuation strain of DA3 a) from 25 to 100 °C for 5 heating-cooling cycles, b) from 25 to 110 °C for 5 heating-cooling cycles, and c) from 25 to 120 °C for 5 heating-cooling cycles. Each test was repeated 3 times. Each data point represents the mean (n = 3), and the error bars represent the standard deviation.

Figure S14. Actuation strain of DA3 upon heating from 25 °C to 110 °C for 1 hour, followed by cooling back to 25 °C.

Figure S15. Actuation strain of DA2 after storage at 80 °C for 1 hour.

Figure S16. a) Thermal stability of DA2 under load at 90 °C, b) thermal stability of DA3 under load at 90 °C. Each test was repeated 3 times.

Figure S17. Recycling of DA2 in DMF and under compression.

Figure S18. Polydomain and monodomain of DA3 dynamic diffraction grating.

Figure S19. a) Images acquired using brightfield unpolarized microscopy during actuation of DA2 dynamic diffraction grating actuator. b) Images acquired using brightfield unpolarized microscopy during actuation of DA3 dynamic diffraction grating actuator.

Figure S20. Experimental and theoretical distances of the diffraction pattern upon heating and cooling cycles. Diffraction occurs from grating lines normal to the nematic director.
